# Rural work in the sugarcane sector and its influences on health: scoping review

**DOI:** 10.47626/1679-4435-2023-779

**Published:** 2023-04-18

**Authors:** Jéssica Cristina Ruths, Pery Francisco Assis Shikida, Isabela Fernanda Larios Fracarolli

**Affiliations:** 1 Saúde Coletiva, Universidade Federal do Paraná, Toledo, PR, Brazil; 2 Agronegócio, Universidade Estadual do Oeste do Paraná, Toledo, PR, Brazil; 3 Escola de Enfermagem, Universidade de São Paulo, Ribeirão Preto, SP, Brazil

**Keywords:** rural workers, working conditions, Saccharum, ethanol, trabalhadores rurais, condições de trabalho, Saccharum, etanol

## Abstract

Since the 2000s, a growing number of studies have been conducted that evaluate rural working conditions in sugarcane plantations. However, there is a need to organize their findings and compile the measures they suggest to protect workers’ health. The objective of this review was to map scientific publications on rural work at sugarcane plantations and its influence on the health of the industry’s workers. The methodological approach adopted was a scoping review, performed according to the Preferred Reporting Items for Systematic Reviews and Meta-Analyses extension for Scoping Reviews checklist. Literature searches were conducted in December 2019 using the Cochrane, Web of Science, PubMed, Scopus, Cumulative Index to Nursing and Allied Health Literature (CINAHL), and Biblioteca Virtual em Saúde databases. The inclusion criteria were original or review studies that answer the research question, with full text available in English, Portuguese, or Spanish, using qualitative or quantitative approaches. Articles were excluded if they did not answer the primary question, if they were duplicates, opinion pieces, theoretical reflections, books, guidelines, theses, or dissertations. A total of 193 studies were identified, 12 of which met the eligibility criteria. These studies showed that sugarcane workers are exposed to thermal, chemical, biological, physiological, mechanical, and emotional risks. The main health problems observed were respiratory, circulatory, renal, and musculoskeletal problems, genotoxic agents, and work-related accidents. It was therefore possible to conclude that the sugarcane work environment can impact workers’ health and disease processes.

## Abstract (pt)

Desde os anos 2000, é crescente o número de estudos que avaliam as condições de trabalho rural da cultura da cana-de-açúcar. Contudo, falta ordenação dos achados e das medidas sugeridas para preservar a saúde dos trabalhadores. Objetivou-se mapear a produção científica sobre o trabalho rural nas culturas de cana-de-açúcar e suas influências sobre a saúde dos trabalhadores da área. Como instrumento metodológico, optou-se pela revisão de escopo, norteada pela *checklist* Preferred Reporting Items for Systematic Reviews and Meta-Analyses extension for Scoping Reviews. O levantamento bibliográfico foi realizado em dezembro de 2019, nas bases de dados Cochrane, Web of Science, PubMed, Scopus, Cumulative Index to Nursing and Allied Health Literature (CINAHL) e Biblioteca Virtual em Saúde. Como critérios de inclusão, os estudos deveriam responder à questão norteadora desta pesquisa, estar disponíveis na íntegra em inglês, português ou espanhol, de abordagem qualitativa ou quantitativa, estudos primários e revisões. Excluíram-se artigos que não responderam ao questionamento principal, que estavam em duplicidade nas bases de dados, artigos de opinião, reflexão teórica, livros, diretrizes, teses e dissertações. Foram encontrados 193 estudos; destes, 12 atenderam aos critérios de elegibilidade e mostraram que os trabalhadores são expostos a riscos térmicos, químicos, biológicos, fisiológicos, mecânicos e emocionais. Os principais agravos à saúde observados foram problemas respiratórios, circulatórios, renais, osteomusculares, genotóxicos e acidentes de trabalho. Portanto, foi possível concluir que o ambiente ocupacional do setor da cana-de-açúcar pode influenciar o processo de saúde e doença dos trabalhadores.

## INTRODUCTION

Brazil is one of the world’s largest producers of sugarcane, accounting for 20% of global production and 45% of global sugar exports. The 2018/2019 harvest yielded 620 million tons of sugarcane, which was used to produce 33 billion liters of ethanol, 29 million tons of sugar, and 21.5 terawatt-hours (TWh) of electricity for the national grid.^1^

The industry plays an important role in generation of income and jobs and, to date, the 2019/2020 harvest has yielded US$ 4 billion in sugar exports and US$ 8 billion in ethanol exports.^1^ According to data from the Centro de Estudos Avançados em Economia Aplicada (CEPEA), in 2017, 3.2% of all Agribusiness employees worked in the sugarcane industry.^2^

The Brazilian scenario is highly conducive to production and exportation of sugar and, in particular, of ethanol, and was constructed within a relationship of the economic and institutional system’s path dependence on fuel alcohol in Brazil. From the mid 1970s onwards, alcohol production was stimulated through an “orchestration” of interests, influenced by distilleries and mills, the machinery and plant industry, the automotive industry, and the State, resulting in a major increase in sugarcane production, with the objective of consolidating fuel ethanol as part of the Brazilian energy mix.^3^

The focus on ethanol extracted from sugarcane was primarily a result of the urgent search for alternative energy sources in response to uncertainty about the future availability of petroleum and environmental issues.^4^ The Brazilian sugarcane industry thus went through a significant process of modernization and diversification, even expanding into regions that had not hitherto produced sugarcane, such as Paraná, Mato Grosso, and Mato Grosso do Sul states, supported by national policies to support the industry.^5^ This phase led to considerable changes in the job market, in selection, organization, and definition of workers’ profiles, and in employment relationships and contracts, with repercussions for society and for the health-disease process of these individuals.^6^

Traditionally, sugarcane production involved preparation of the soil, choice of cultivars, planting, fertilization, and conservation of the soil. Harvesting includes cutting the cane, loading it, and transporting it for processing in mills and distilleries.^7^ Cane can be harvested manually or mechanically. In Brazil, approximately 56% of the 2009/2010 crop was harvested mechanically, without burning the cane beforehand. For the 44% that was harvested manually, pre-burning was used to make it easier to cut and increase productivity.^8^ However, by 2019, around 98% of harvesting in the Mid-South of Brazil had already been mechanized.^1^

However, despite the technological development achieved in the sugar and alcohol industry, over recent years the working conditions and occupational health of its workers have been attracting attention. Growth of the sugarcane agroindustry intensified use of manual labor, encouraging migration from poorer regions of the country, in search of work during the sugarcane harvests.^7^ In both manual and mechanized harvesting, workers can be subjected to long work shifts in environments in which they are potentially exposed to risks^5,6,9-11^ and they may also be induced to exert themselves excessively by production-based payment systems.^6,7,9^

Since the 2000s, a growing number of studies have been conducted that evaluate rural working conditions in sugarcane plantations. However, there is a need to organize their findings and compile the measures they suggest to protect workers’ health. In view of the importance of the sugarcane agroindustry as a source of income and employment, the findings of this review will be useful to demonstrate the implications of the industry’s rural activities for occupational health.

Therefore, working from the premise that the health and disease process is influenced by health determinants, one of which is the working environment, the objective of this study was to map scientific publications on the process of rural work at sugarcane plantations and its influence on the health of the industry’s workers.

The analyses presented in this paper are based on the conceptual framework of the social-ecological theory, which holds that an individual’s conditions of health and wellbeing are determined by interactions between physiological, psychological, social, environmental, political, and cultural factors, making it a model of theoretical principals for understanding the interrelations between the factors that comprehend the health and disease process.^12,13^

## METHODS

This is a qualitative study adopting the scoping review methodological approach, conducted according to the Preferred Reporting Items for Systematic Reviews and Meta-Analyses extension for Scoping Reviews (PRISMA-ScR) checklist. The PRISMA-ScR comprises 22 items that cover title, abstract, introduction, methods, results, discussion, and financing, which incorporate recommendations for conducting scoping reviews proposed by the Joanna Briggs Institute (JBI).^14^ The tool helps to clarify research areas, identify knowledge gaps, map the most important concepts related to a given subject.^14^

The review comprised the following steps: (a) formulation of the research question; (b) identification of relevant studies; (c) selection of studies; (d) mapping the data; and (e) grouping, summarizing, and reporting of results.^14^

The Population (P), Concept (C) and Context (C) (PCC) strategy was used to formulate the research question,^14^ where P is sugarcane workers; C the rural work process; and C health conditions. Based on these definitions, the question this study seeks to answer was formulated as follows: “How does the rural work process of sugarcane cultivation affect the conditions of workers’ health?”

Literature searches were conducted in December 2019 using the Cochrane, Web of Science, PubMed, Scopus, Cumulative Index to Nursing and Allied Health Literature (CINAHL), and Biblioteca Virtual em Saúde databases. Searches were conducted independently by two researchers and no time or space limits were imposed. Keywords were selected from the Health Science Descriptors (DeCS) thesaurus and combined with each other using Boolean operators, as follows: “Workers” “AND” “Working Conditions” “AND” “Saccharum” “OR” “Ethanol”. All keywords were employed in English.

The Cochrane database identified 63 studies; Web of Science found 19; PubMed returned 25; Scopus listed 36; CINAHL retrieved 30; and the Biblioteca Virtual em Saúde identified 20 studies. The inclusion criteria were qualitative or quantitative original research or systematic reviews, meta-analyses, or scoping reviews that answer the research question, with open-access full text available in English, Portuguese, or Spanish via the chosen databases. Articles were excluded if they did not answer the research question, if they were duplicates, opinion pieces, theoretical reflections, books, guidelines, theses, or dissertations.

After the initial searches, the titles, abstracts, and keywords were analyzed. Articles selected were then read in full and data were extracted using a modified version of the Marziale integrative review data collection instrument.^15^

Data were analyzed and selected by the lead researcher and confirmed by the second researcher. Any queries and contradictions were examined and evaluated by all authors.

## RESULTS

**Figure 1:**
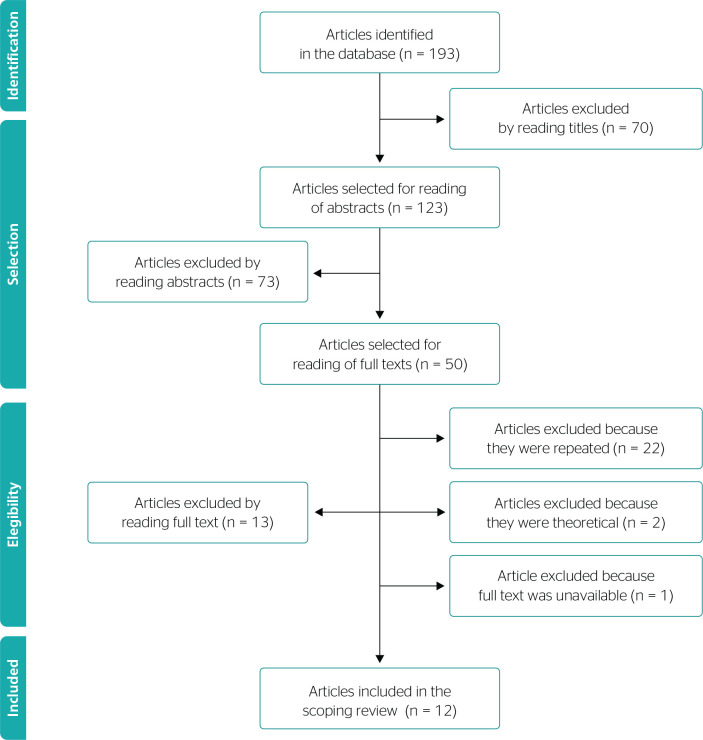
Preferred Reporting Items for Systematic Reviews and Meta-Analyses extension for Scoping Reviews (PRISMA-ScR) flow diagram illustrating selection of articles from databases, 2021. *Articles excluded because they did not answer the research question.

A total of 193 studies were found initially and after reading their titles and abstracts, 50 were selected for reading in full. Twenty-two were excluded because they were listed on more than one database, 1 because the full text was unavailable, 2 because they were theoretical studies, and 13 because they did not meet the eligibility criteria, only dealing with creation of jobs and income in the sugarcane industry. The final sample for the review comprised 12 articles. The process of searching for and selecting articles is illustrated in a flow diagram (Figure 1).

The 12 articles included in the review were published from 2006^16^ to 2018.^5-22^ Nine were conducted in Brazil,^4-7,10,17-20^ 1 in Guatemala,^21^ and 1 in El Salvador.^22^ All of them deal with the population of sugarcane harvest workers, with similar objectives related to working in this agricultural activity, as shown in Table 1.

**Table 1:** Characteristics of the studies included in the review, by author, year, study location, study type, sample, objective, results, and answers to the research question

Author(s), year, study location	Type of study and sample	Objective	Results	Answers to the research question
Penteado et al.,^17^ 2013, Brazil	Quantitative, cross-sectional, exploratory study with 50 sugarcane cutters	To identify work accidents during manual sugarcane cutting at a mill in the Northwest of São Paulo	40% of the interviewees had had a work accident; machetes were responsible for 90% of accidents; lower limbs were involved in 45% of accidents; 90% of workers wore PPE; mechanization of the harvest reduced the number of accidents, but increased their severity.	Sugar cane cutters are subject to an intense work rate and repetitive movements that force them to adopt unhealthy musculoskeletal positions, predisposing them to occupational risks; migrants’ living conditions are unhealthy.
Bodin et al.,^22^ 2016, El Salvador	Longitudinal, descriptive and quantitative study, with 60 sugarcane workers	To evaluate the feasibility of delivering an intervention based on the OSHA “Water. Rest. Shade.” (WRS) program while cutting cane to avoid stress caused by heat and dehydration, without reducing productivity	Water consumption increased by 25% after the intervention and symptoms of heat stress and dehydration reduced; individual daily production increased from 5.1 tons to 7.3 tons of cane.	The study identified repetitive movements, heat exposure, and likelihood of dehydration, which could be related to prevalence of chronic kidney disease of unknown cause and musculoskeletal disorders; workers were wearing PPE; access to water, rest, and shade during the working day provided workers with considerable relief from the high level of heat stress.
Luz et al.,^6^ 2012, Brazil	Longitudinal, descriptive and quantitative study, with 30 migrants	To characterize the working conditions of migrant sugarcane workers and assess their body composition from start to end of the harvest in Piracicaba, São Paulo	Over the course of the harvest, creatine kinase rose and there were significant (P < 0.001) reductions in body fat and weight in the first half of the harvest, more pronounced in migrants.	The study identified few rest breaks during the working day and exposure to heat stress. The work can cause a small increase in muscle mass; payment per production may impede workers from taking breaks and regulating work loads, provoking exacerbation of physical overload.
Leite et al.,^5^ 2018, Brazil	Review study including 52 articles	To describe the main work risks for sugarcane cutters and their effects on workers’ health.	A total of 89 articles were identified, 52 of which met selection criteria and were selected for the review.	The studies showed that sugarcane cutters work in conditions that cause physical, mental, and thermal overload, exposure to pollutants, and vulnerability to accidents. The main effects observed were respiratory, cardiovascular, renal, musculoskeletal, heat stress, dehydration, genotoxic, and caused by accidents.
Minayo-Gomez,^20^ 2011, Brazil	Documentary analysis of documents from institutions and discussion forums covering proposals for interventions in the working conditions in the sugar and alcohol industry	To analyze the dynamics of the construction of forms of inter-sectoriality in practices aimed at improving working and living conditions of workers in the sugarcane and alcohol industry	Appropriation of strategic knowledge produced by researchers studying the sugarcane industry can be an instrumental resource for legal actions, monitoring, and surveillance.	Sugarcane cutting work is a repetitive activity, involving exposure to high temperatures, soot, and dust; workers lose significant weight and body fat during the harvest. Work intensity can be increased by payment per production; in general, women are allocated to jobs such as “pituca” (scavenging pieces of cane and clearing stones).
Rocha et al.,^10^ 2010, Brazil	Exploratory and quantitative study with 39 sugarcane workers	To analyze how work and life situations can cause risks to the health of workers involved in manual and automated sugarcane cutting	The study found exposure to solar radiation, rain, wind, dust from the soil, soot from cane burning, high temperatures, pesticides residues, and venomous animals. The main impacts were respiratory, musculoskeletal, and psychological problems and work-related accidents. There was a predominance of migrants from the Northeast.	Manual cutting: workers perform heavy and repetitive movements continually for their 8-hour shifts, which leads to exhaustion. They wear PPE. Payment is per production. Carriers are subject to accidents involving machetes and venomous animals. Respiratory problems were related to burning the cane. Mechanized harvest: harvester operators receive a fixed wage, with a 10-hour shift. Harvester cabins are closed, have air-conditioning, and seats are adjustable for height, with elbow rests. Operators only stop working when the harvester breaks down or for maintenance, cleaning, and changing the cutting knives. Back problems were related to long periods sitting. Operators are involved in higher risk accidents, related to maintenance of harvesters, falls, collisions with trucks, and contact with the electrical system.
Barbosa et al.,^18^ 2012, Brazil	Quantitative panel study with 28 sugarcane cutters	To assess the occurrence of cardiovascular diseases and possible mechanisms associated with burnt sugarcane harvesting involved in these events	Compared to the pre-harvest period, blood pressure and blood coagulation increased.	The study observed physical and thermal overload and exposure to higher levels of particulate material; WBGT was higher than limits recommended for continuous work. Muscle injuries, blood coagulation changes, heart rate variability, systemic oxidative stress, and increased blood pressure were observed. It was not possible to assess the individual impact of each isolated risk factor on the changes observed.
Ruddy et al.,^19^ 2012, Brazil	Cross-sectional, descriptive and quantitative study. Sample not reported	To present an intervention proposal based on implementation of a proven methodology involving workers and the people responsible for planning and managing production	It was found that 20% of workers would suffer an upper limb injury over a 5-to-10-year period.	The work process involves repetitive movements demanding strength. The right arm is more often affected, because of use of the machete.
Dally et al.,^21^ 2018, Guatemala	Longitudinal, descriptive and quantitative study with 4,095 sugarcane cutters	To evaluate the relationship between temperature exposure, kidney function, and productivity.	There was a 1.16 (95%CI 2.87-0.54) ton reduction in sugarcane cut among workers exposed to temperature 34 ºC WBGT compared with those exposed to 29 ºC. Workers with impaired kidney function were twice as likely to leave employment (HR: 2.92, 95%CI 1.88-4.32).	Heat extremes may be associated with loss of agricultural worker productivity, especially among those with impaired kidney function.
Carvalho-Junior et al.,^7^ 2012, Brazil	Longitudinal, descriptive and quantitative study with 44 sugarcane cutters	To assess health-related quality of life in sugarcane cutters.	There was a reduction in quality of life measured by questionnaire, after the harvest.	Health-related quality of life in sugarcane cutters was reduced after the harvest period in the vitality domain. Workers who remained working during harvest were those with lower scores for social aspects.
Rocha et al.^4^, 2007, Brazil	Exploratory quantitative study, with 39 sugarcane cutters	To identify the individual, social, and environmental factors predisposing the manual sugarcane workers to illnesses.	Physical effort, hectic work rhythm, intense heat, dust, and venomous animals were the determinants of illness.	Manual sugarcane cutting can expose workers to high temperatures, agrochemical residues, physical overload, and musculoskeletal problems related to the frenetic work rate. Respiratory problems were associated with exposure to dust and soot. Injuries are caused by handling of machetes and exposure to venomous animals. Workers reported physical and psychological exhaustion. Payment is per production. Lodgings house from 8 to 10 workers and were simple constructions with access to sanitation.
Bosso et al.,^16^ 2006, Brazil	Case-control study with 39 sugarcane workers	To assess urinary levels of 1-hydroxypyrene in sugarcane workers during harvest and during the non-harvest period, as a biological marker of exposure to polycyclic aromatic hydrocarbons	Workers exposed to polycyclic aromatic hydrocarbons had urinary 1-hydroxypyrene levels nine times higher than those not exposed (p < 0.10).	Workers were exposed to genotoxic agents, mutagenic agents, and polycyclic aromatic hydrocarbons during harvest. There are indications of potential risk of respiratory diseases and/or lung cancer.

PPE = personal protective equipment; HR = hazard ratio; WBGT = Wet Bulb Globe Temperature; OSHA = Occupational Safety and Health Administration; WRS = work-related stress.

In all, 8 articles were published in English^5,6,10,16,18,19,21,22^ and 4 in Portuguese.^4,7,17,20^ With regard to study design, there was one literature review,^5^ one documentary analysis,^20^ two cross-sectional descriptive studies,^17,19^ four descriptive longitudinal studies,^6,7,21,22^ two exploratory studies,^4,10^ one panel study,^18^ and one case-control study.^16^

The articles included aimed to assess quality of life of sugarcane workers,^7^ how working conditions influence workers’ body composition,^6^ occurrence of cardiovascular diseases,^18^ the influence of exposure to polycyclic aromatic hydrocarbons,^16^ the risk of exposure to excessive heat,^4,22^ and occupational accidents in the industry.^5,16-22^

Risks identified that affect sugar and alcohol industry workers included thermal (high temperatures),^4,22^ chemical (soot and agrochemical residues),^5,16^ biological (venomous animals), mechanical (accidents),^5,20^ physiological (repetitive and strenuous movements, long shifts, unhealthy postures, inadequate hydration),^6,10,18,21,22^ and emotional factors (frenetic work rate, payment per production, few scheduled breaks, migration, poor living conditions).^6,17,18^ These risks may lead to respiratory,^16^ circulatory,^5,18^ renal,^21^ and musculoskeletal problems,^4,10,18,19^ weight loss,^6^ genotoxic^5,16^ and accidents.^4,5,10^

It should be pointed out that Bodin et al.^22^ conducted the only intervention in the cane cutting process, finding that personalized provision of cool fresh water and scheduling breaks in areas with portable shades was sufficient to increase water intake by 25%, reducing symptoms of heat stress and dehydration and increasing individual daily production from 5.1 to 7.3 tons of cane per person.

## DISCUSSION

The 12 articles included in this review deal with the relationship between the process of working in the sugar and alcohol industry with workers’ sickness. It was found this work involved a high rate of work,^4,6,17,18^ repetitive movements,^10,19^ intense physical exertion,^4,6,10,18,21,22^ heat exposure,^4,10,21,22^ and other risk factors, which can be detrimental to body weight,^6^ increase blood pressure,^18^ cause musculoskeletal injuries,^4,10,18,19^ lung cancer,^16^ and other respiratory diseases,^4,16^ cause accidents,^4,5,10^ and reduce quality of life.^7^ Other studies also associated health problems with rural sugarcane work.^23-25^

It takes several months to grow sugar cane and it is necessary to hire large numbers of workers, primarily for manual harvesting. Luz et al.^6^ and Barbosa et al.^18^ highlight the predominance of migrants workers from the Northeast of Brazil. The predilection for this source of labor is related to the fact that the labor is cheaper and these workers have the commitment to work in these conditions. However, these migrants seek employment cutting sugarcane because they are expelled from their own regions by lack of employment and the responsibility to provide for their families’ subsistence, since they are the heads of their families.^26,27^

In general, despite the living and working conditions, Galiano et al.^27^ report that the workers intend to return to work on future harvests, because migration to cut sugarcane was one of the few employment options they found.

The work process may be based on manual or mechanical sugarcane cutting. The choice of method is dependent on the costs incurred and the possibilities for use of mechanization. Mechanized areas have a slope less than or equal to 12% in regions with more than 150 hectares and the soil enables use of mechanization for harvesting. Non-mechanizable land has slopes exceeding 12% and soils that do not enable use of mechanization.^28^

Manual cutting may be preceded by burning. Sugarcane is burnt to eliminate leaves, enable better access to the cane tips, and reduce the risk of accidents involving venomous animals.^17^ However, the practice causes emission of particulate material that, when inhaled, can affect the upper and lower airways, causing symptoms of respiratory diseases among the workers.^4,5,10,16^

Suspension of the particulate material present in sugarcane and the soil is released by the machete blows and can be witnessed by the blackened color of the workers’ clothes during harvest. Prado et al.^9^ found a higher prevalence of respiratory symptoms and reduced pulmonary function among sugarcane cutters compared to a reference population.

However, initiatives are in course to minimize the effects of sugarcane burning. São Paulo State Law 11.241/2002 demanded that by 2006, only 30% of areas with slopes less than 12% could be burnt and stated that from 2021 onwards, sugarcane burning would be entirely illegal.^11,28^

Also with regard to manual harvesting, the working week lasts 5 working days.^7,10,19^ Shifts start at 7 a.m. and end at approximately 4 p.m..^7,10,19^ A day’s harvest parcel is generally five to six lines wide and 100 to 150 meters long, which can vary depending on each cane cutter’s speed and physical strength.^4,6,22^ Workers must cut the cane close to the foot, a few centimeters above the ground, where sucrose concentration is highest, swinging the machete at the level of the shoulder while bending at the knees and/or a waist. The cut stems are lifted and carried in bundles weighing around 10 to 15 kg for 2 to 5 meters, to be arranged in lines and taken away on trucks.^5,10,17,22^ Cutting close to the ground should be done carefully, so that the roots are not affected, compromising regrowth.^17^

After the sugarcane has been cut, the straw is removed with sequential movements involving spinal flexion and forming an angle close to 90 degrees to the lower limbs to reach the ground.^6,10,17,19^ Minayo-Gomez^20^ points out that, in general, this job, known as “bituca”, is assigned to women, because of the physical strength needed for sugarcane cutting. The last step is to load the sugarcane and transported it to the mills for processing.

At the end of the harvest, production is counted. According to the articles analyzed, production can vary from 6 to 14 tons/man/day, which involves an average of 3,100 spinal flexions, 3,500 machete blows, and 1,000 spinal column rotations.^5,17,18^ The movement cycles last approximately 5.6 seconds, which is considered a physical risk, since cycles lasting less than 30 seconds are a risk for osteoarticular injuries.^20^

This work demands a combination of physical strength and resistance for cutting and carrying cane form the fields to the trucks. The movements and positions involved in this rural work can predispose to repetitive stress injury, significantly reduce body weight, waist circumference, and body mass index (BMI), increase blood pressure, and lead to musculoskeletal diseases.^4,5,17-19^

The intense workload was observed by Carvalho Junior et al.,^7^ analyzing the quality of life of sugarcane cutters, finding reduction in vitality domain scores in the middle and at the end of the harvest. Confirming this, Vilela et al.^29^ observed that the frenetic work rate and intense physical effort increased cardiovascular load and reduced body weight and body fat.

Another risk associated with sugarcane cutting is accidents. The majority of accidents involve machetes and the body sites most often involved are the legs, hands, and feet.^17,10^ In these cases, the agent of injury is the worker’s tool. Teixeira & Freitas^30^ also observed that machetes were the agent involved in 49% of work accidents among sugarcane cutters in Sâo Paulo state.

Moreover, physical exhaustion, exposure to sunlight, and wearing multiple layers of clothes to protect from ultraviolet (UV) light can lead to heat overload and stress.^5,6,10,18,20^ The Ministry of Labor and Employment’s Regulatory Standard 15^31^ requires 15 minute breaks every 45 minutes when WBGT temperatures exceed 30ºC for mild activities, 26.7ºC for moderate activities, and 25ºC for heavy activities. Work requiring intense physical effort, as is the case with sugarcane cutters, cannot be performed in temperatures exceeding 30ºC.^29^ Barbosa et al.^18^ identified WBGT temperatures higher than 28.4ºC, exceeding the recommended limit for continuous activities. It should be emphasized that spending time in unhealthy temperature conditions can cause heat stress, leading to deterioration in general health status, psychosensory changes and reduced production.^32^

Since sugarcane is harvested in direct sunlight, the need for hydration is constant. Cutters bring water to the fields in containers that are intended to last the whole day, which could cause reduced water consumption. Reduced water consumption can cause dehydration, heat stress, and volume depletion - known risk factors for kidney disease.^22,33^ Bodin et al.^22^ point out that symptoms associated with exhaustion (fever, nausea, and cramps) and dehydration (very dry mouth and little urine) reported by sugarcane cutters reduced substantially after water intake was increased. Wesseling et al.^34^ assessed kidney function in 29 sugarcane cutters in Nicaragua, observing a significant fall in kidney function over 9 weeks of work. Dally et al.^21^ point out that workers with reduced kidney function may have reduced work productivity.

With regard to breaks, workers have a 1 hour lunch break and approximately two 15 minute rest breaks^7,10,18,19^ at other times. However, breaks tend not to be respected because of payment per production.^19^ The small number of breaks is also unfavorable for muscles to recover and for electrolyte replacement, which can cause harm to physical integrity over the medium and long term.^6^

Manual cutters are paid per production. This mechanism encourages workers to work faster, in order to earn more and increase the likelihood of being hired for subsequent harvests.^5,10,20^ Regulatory Standard 17, related to ergonomics,^35^ states that payment per production can only be employed if it does not involve risk of health. With regard to the sugarcane industry, Laat^25^ has highlighted the importance of suppressing this method of remuneration.

To protect themselves from risks, workers wear hats and scarfs, to protect their face and neck, and two overlapping long sleeved shirts. The employers provide other items of personal protective equipment (PPEs) for the cutters, such as glasses, gloves, leather leg guards, and steel-capped boots.^4,10^ Care should be taken with the quality of these PPEs, since gloves that slip and opaque safety glasses can be aggravating factors in accidents with machetes and venomous animals.^20^

With regard to mechanized harvesting, it was observed that a single shift lasts 10 hours; half of the harvesting is done during day shifts and half at night. Operators only pause work for repairs or maintenance to the harvesters, such as for provisioning and changing cutting knives and when they overheat. These workers are paid a fixed wage and the majority of them do not know how much sugarcane their machines cut.^10^ They thus work at an intense rate, targeting high productivity, optimization of resources, reduction of costs, and profitability.

The harvester cabins are closed, have air-conditioning, and the seats are height and adjustable and reclinable, with adjustable elbow supports. The operator controls cutting with pedals and levers. The pedals are on the cabin floor, forming a 90 degree angle with relation to the legs, while the levers are in front of the seat, between the operators legs and on the right hand side. The operator uses the left hand to control the central levers and the right hand to work the levers on the right and must pay constant attention to the land, to follow the correct path, and to the side, to monitor the overload truck. It is also necessary to pay great attention to the control panels that show the condition of temperature, oil, rotation, and velocity.^10^

The most obvious risks to operators are injuries, respiratory and circulatory problems, and high blood pressure resulting from collisions, falls, contact with the electrical system, venomous animals, exposure to dust and soot, long shifts spent sitting, and repetitive movements.^10^

Both the manual and mechanical cutting workers may be exposed to chemical and psychological risks. With regard to chemical risks, Bosso et al.^16^ found that sugarcane workers were significantly more exposed to genotoxic agents, mutagenic agents, and polycyclic aromatic hydrocarbons, and were more subject to lung cancer and respiratory problems. Martinez-Vanezuela et al.^36^ assessed chromosomal damage in burnt sugarcane workers in Sinaloa, Mexico. The results revealed higher rates of micronuclei in exposed subjects compared to non-exposed people, which could indicate a risk of cancer and pulmonary problems.

The psychological risk is imposed by the intense work rate, incentivized by payment per production, risk of accidents, the need for constant attention and concentration to avoid accidents, low pay, and, in the case of migrant workers, absence from the family, distance from home, and poor living conditions.^4,10,17^

Strategies for protection of the health of rural sugarcane workers suggested in the articles analyzed were reassessing the quantity and distribution of rest breaks, maintaining the machetes sharpened, instruction on more comfortable postures, fulfilling ergonomic principles, and increased water intake and quality.^17,19,22^

Strong points of the studies reviewed include direct observation of the working^6,10,17,18,21,22^ and living conditions^4,6,10^ of the industry’s workers. There was an analysis of the effect of heat and kidney failure on the productivity of a large sample of 4,095 workers.^21^ One study implemented an intervention in the work process, showing that introduction of breaks and availability of water and shade helped to improve the conditions for workers’ health and increased production.^22^

With regard to limitations, one study analyzed the work environment and work organization by interviewing workers only, with no on-site observation or observation of work activities.^7^ One study did not report the sample size.^19^ There was also a discussion of the difficulty of assessing the impact on the problems observed of each risk factor in isolation (air pollution, heat, and effort).^18^

## CONCLUSIONS

This review, employing analyses based on the social ecological theory was able to identify occupational determinants of the health and disease process that can impact on the health of sugarcane industry workers, which were high temperatures, soot, agrochemical residues, venomous animals, occupational accidents, repetitive movements, unhealthy positions, low water intake, long work shifts, frenetic work pace, payment per production (which tends to intensify the manual cutting process), few rest breaks, migration, and poor living conditions. These factors can cause respiratory, circulatory, renal, musculoskeletal, and genotoxic problems, raised blood pressure, and work accidents.

Therefore, multidimensional interventions in many different determinants of the health and disease process of these workers are axiomatic if true health promotion is to be achieved.

Finally, limitations of this review include a lack of methodologically robust studies, of investigations of the sugarcane cultivation work process on the international level, and also of studies investigating mechanized sugarcane cutting. It is suggested that future studies should extend longitudinal assessments of the working conditions and activities of all of the workers, covering the harvest and pre-harvest, and expand analyses of the occupational risk factors of mechanized cutting workers.

In view of the importance and representativeness of the sugarcane industry as a source of income and employment, it is hoped that the results of this review will demonstrate the importance of studying occupational situations that can impact on the health and disease process, contributing data that identify vulnerable groups, that shed light on how the industry works, and that help managers to develop strategies for prevention of occupational injuries.
